# Superficial Siderosis of the Central Nervous System: A Report of Two Cases With Spinal Pathology and a Review of the Literature

**DOI:** 10.7759/cureus.60486

**Published:** 2024-05-17

**Authors:** Jing Chen, Philippines Cabahug, Travis Edmiston

**Affiliations:** 1 Rehabilitation Medicine, Singapore General Hospital, Singapore, SGP; 2 Physical Medicine and Rehabilitation, Johns Hopkins University School of Medicine, Baltimore, USA; 3 Physical Medicine and Rehabilitation, International Center for Spinal Cord Injury, Kennedy Krieger Institute, Baltimore, USA

**Keywords:** thoracic myelopathy, spinal surgeries, tethered cord syndrome, gunshot wound, superficial siderosis

## Abstract

Infratentorial superficial siderosis, characterized by hemosiderin deposition in the subpial layers of the brainstem, cerebellum, and spinal cord, is a rare progressive neurologic disorder. We present two cases of infratentorial superficial siderosis. Case 1 involves a 62-year-old female previously diagnosed with tethered cord syndrome and thoracic myelopathy, who, following 11 spinal surgeries, presented with worsening myelopathy, hearing loss, and cognitive impairment. Brain magnetic resonance imaging (MRI) revealed extensive superficial siderosis affecting the cerebellar vermis and bilateral cerebellar hemispheres. Case 2 is a 27-year-old male with a traumatic T4 spinal cord injury from a gunshot wound, complicated by a syrinx, experiencing persistent lower back pain and lower limb spasticity. MRI confirmed superficial siderosis in the spinal cord. This case report explores the clinical manifestations, imaging findings, management strategies, and prognosis of these cases. It also highlights the diverse clinical presentations and underlying etiologies of infratentorial superficial siderosis. It emphasizes the pivotal role of MRI with iron-sensitive sequences for definitive diagnosis. Furthermore, the management underscores the significance of a multidisciplinary team approach in providing comprehensive care for affected individuals.

## Introduction

Superficial siderosis (SS) of the central nervous system is a rare condition, with reported prevalence rates ranging from 0.05% to 0.14% [1-3]. It is a chronic disorder characterized by the deposition of hemosiderin in the subpial layers of the brain and spinal cord, attributed to chronic low-grade bleeding into the subarachnoid space [4]. The disorder is typically classified into two main types based on the affected brain regions: cortical SS (cSS) and infratentorial SS (iSS) [5,6]. iSS specifically involves hemosiderin deposition primarily in the brainstem, cerebellum, and occasionally, the spinal cord.

The typical clinical manifestations associated with iSS include slow progressive hearing impairment, cerebellar symptoms, and myelopathy-related symptoms [4,6]. Owing to the rarity of this condition, it is often under-recognized and the confirmation of the diagnosis is frequently delayed. Herein, we report two cases illustrating iSS, discussing their clinical manifestations, image findings, management strategies, and prognosis.

## Case presentation

Case 1: SS in a case with multiple spinal surgeries

A 62-year-old female with a medical history of reflex sympathetic dystrophy (RSD) initially presented 27 years ago with bilateral lower limb weakness and sensory deficits following the implantation of an epidural morphine pump for pain management of her RSD. Further evaluation revealed granuloma formation at the morphine pump tip resulting in thoracic spinal cord compression. Surgical removal of the morphine pump and decompression of the spinal cord led to improvement in her lower limb weakness and sensory impairment. However, four years later, she reported recurrent bilateral lower limb weakness and bladder dysfunction. Spinal magnetic resonance imaging (MRI) indicated a tethered spinal cord at T7-T8 with thoracic myelopathy. Thoracic laminectomy and untethering resulted in subsequent improvement in neurological symptoms. Nevertheless, six years later, her lower limb weakness, sensory deficit, and bladder dysfunction recurred, with the myelogram CT revealing tethering at the previous site at T7 and T8. She underwent revision laminectomy, untethering of the spinal cord, and duraplasty. Despite transient improvement, symptoms recurred after a few months, leading to repeated revisions of laminectomy, untethering of the spinal cord, and duraplasty. She underwent a total of 11 spinal surgeries, including laminectomy and fusion, untethering of the spinal cord, and spinal duraplasty at her thoracic region.

Subsequently, she noted a gradual escalation in weakness affecting both her arms and legs, accompanied by frequent instances of dropping objects, and right-sided facial numbness. She experienced bilateral progressive hearing loss, and reported worsening memory loss, prompting further investigation with an MRI of the brain, cervical, and thoracic spine.

**Figure 1:**
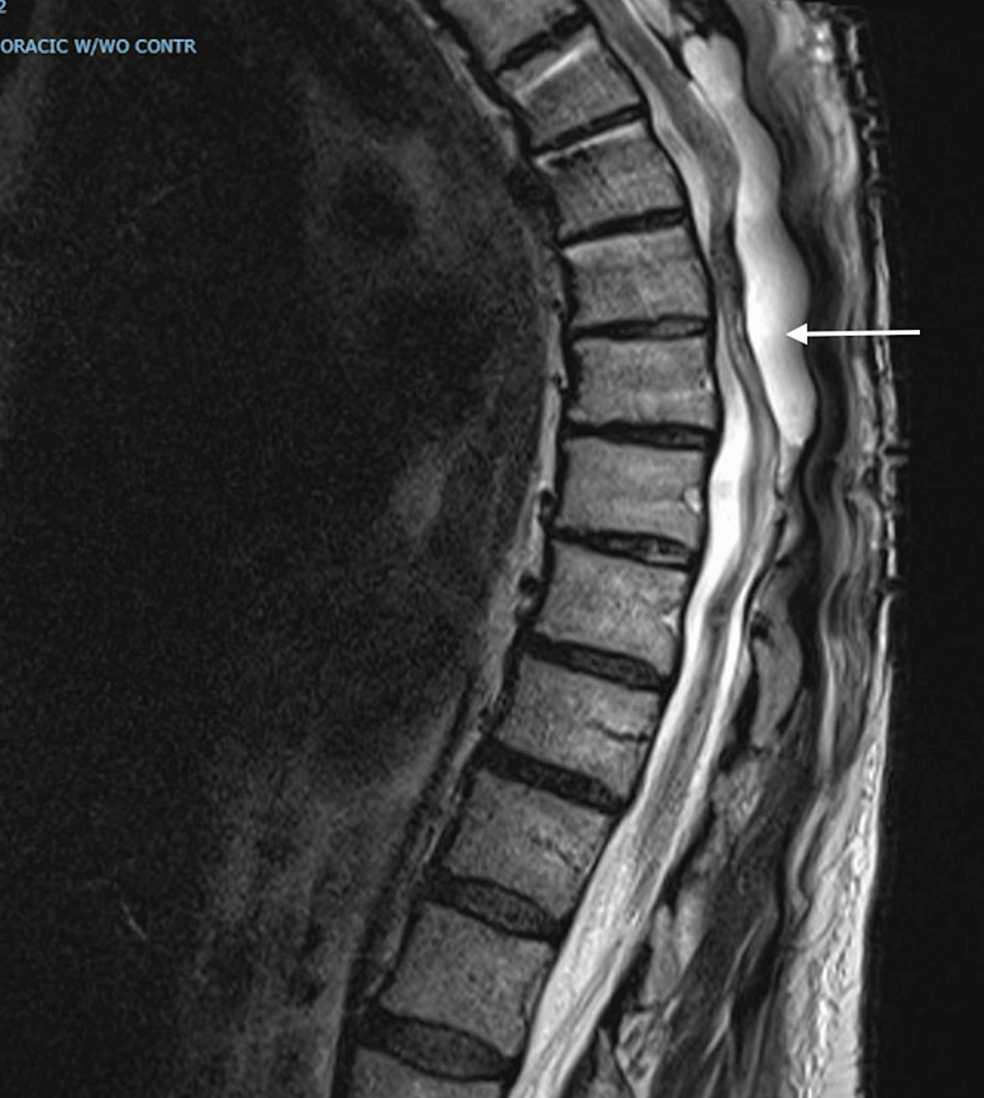
Case 1: MRI of the thoracic spine Epidural collection extending from T5 to T9 level and measuring 1.4 cm anteroposterior and 9.2 cm craniocaudal (arrowed). Unchanged appearance of T5-T10 region dorsal epidural collection, myelomalacia, and postoperative changes.

The MRI of her thoracic spine revealed an unchanged appearance of myelomalacia, postoperative changes, and an epidural collection extending from T5 to T9, measuring 1.4 cm anteroposterior and 9.2 cm craniocaudal (Figure 1).

**Figure 2:**
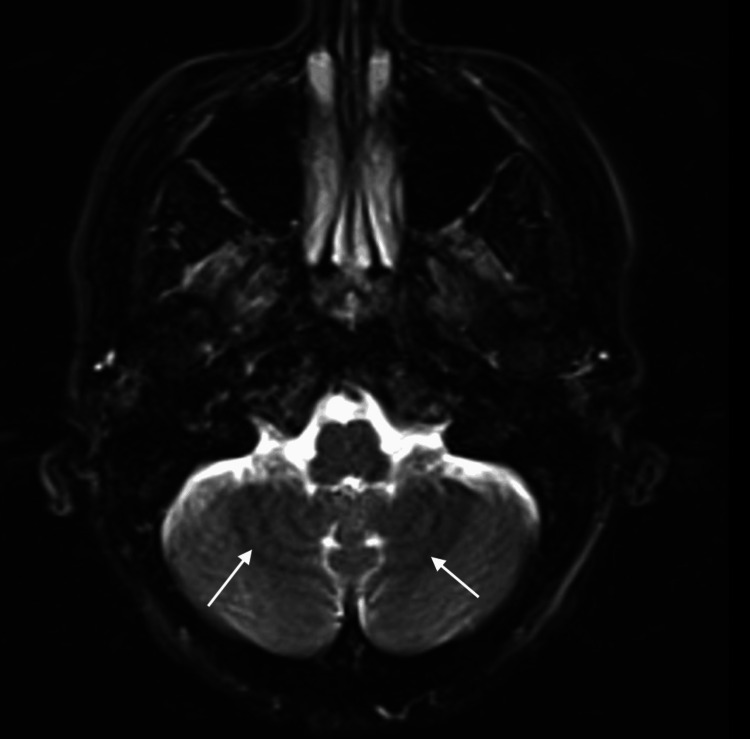
Case 1: MRI of the brain, T2-weighted image The hemosiderin deposition was not clearly visualized on conventional T2-weighted MRI images (arrowed).

**Figure 3:**
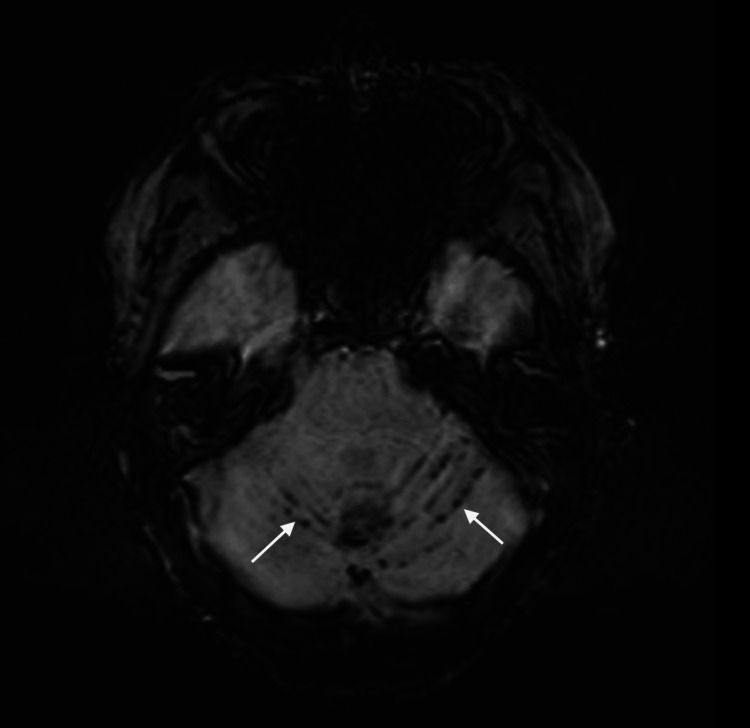
Case 1: MRI of the brain, susceptibility-weighted image (SWI) SWI confirms hemosiderin deposition involving the cerebellar vermis and bilateral cerebellar hemispheres (arrowed).

The MRI brain showed extensive SS, prominently involving the cerebellar vermis and bilateral cerebellar hemispheres. The hemosiderin deposition was not clearly visualized on conventional T2-weighted MRI images, but it is apparent on susceptibility-weighted images (SWIs) (Figures 2-3).

Her condition was managed conservatively through a multidisciplinary team approach. She was referred to a neuropsychologist for cognitive assessment. She was also provided with a hearing aid by Audiology. Skilled physical and occupational therapy was administered to improve her balance, gait, and upper limb function.

Case 2: SS in a case with spinal cord injury (SCI) from a gunshot wound

A 27-year-old male with no significant medical history presented at the Emergency Department in 2021 for multiple gunshot wounds resulting in cardiac arrest. His injuries comprised bilateral hemopneumothorax requiring bilateral chest tube placement, a right rib fracture, and a T2-T3 vertebral fracture leading to partial spinal cord transection. An initial MRI of the thoracic spine revealed open fractures of T2 and T3, along with an associated syrinx and signal changes within the cord at those levels. His International Standards for Neurological Classification of Spinal Cord Injury (ISNCSCI) exam confirmed a T4 American Spinal Cord Injury Association (ASIA) Impairment Scale B SCI [7]. The spinal fractures were managed conservatively with a thoracic lumbar sacral orthosis (TLSO) for three months.

**Figure 4:**
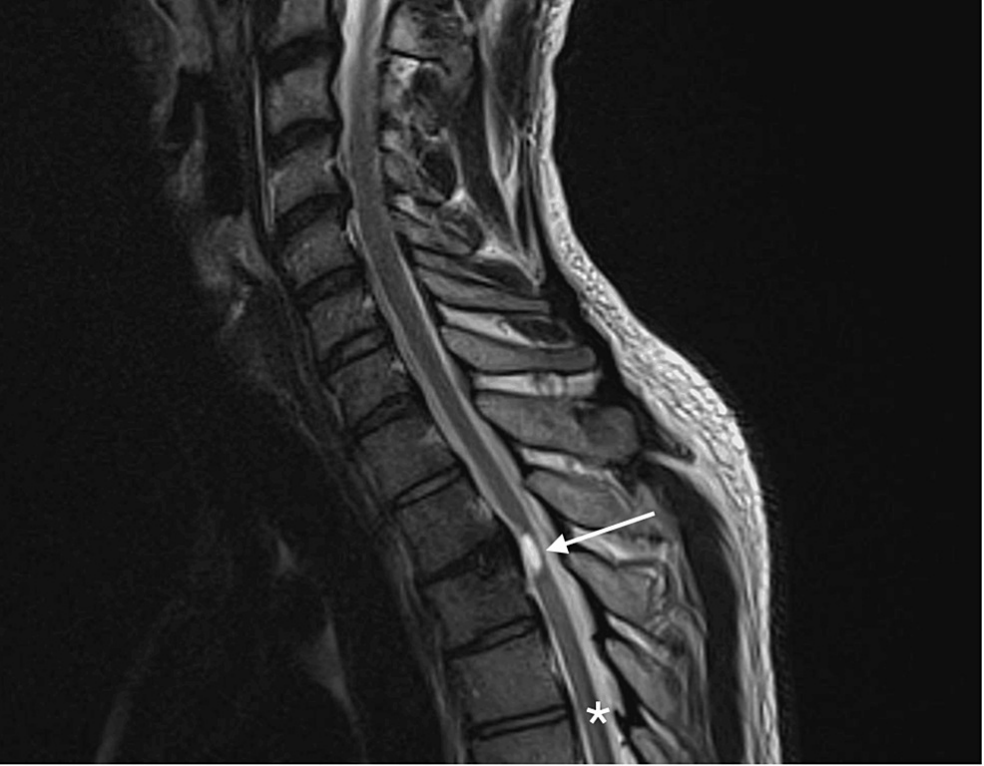
Case 2: MRI of the spine Stable syrinx and parenchymal volume loss around the syrinx at T2-T3 (arrowed). Superficial siderosis is partially visualised at T4-T5 (star).

One year after the initial injury, he reported lower back pain accompanied by increased spasms over bilateral gluteal regions and extending to his bilateral lower extremities. He was started on baclofen at 20mg four times daily and tizanidine at 4mg four times daily to address the spasms. Additionally, he received botulinum neurotoxin type A injection to his lower limbs, targeting the bilateral hip adductors, bilateral gastrocnemius, and bilateral soleus. Despite these interventions, he noted minimal improvement in his spasms. Subsequent MRI scans of the entire spine revealed stable syrinx and parenchymal volume loss around the syrinx at T2-T3 (Figure 4).

**Figure 5:**
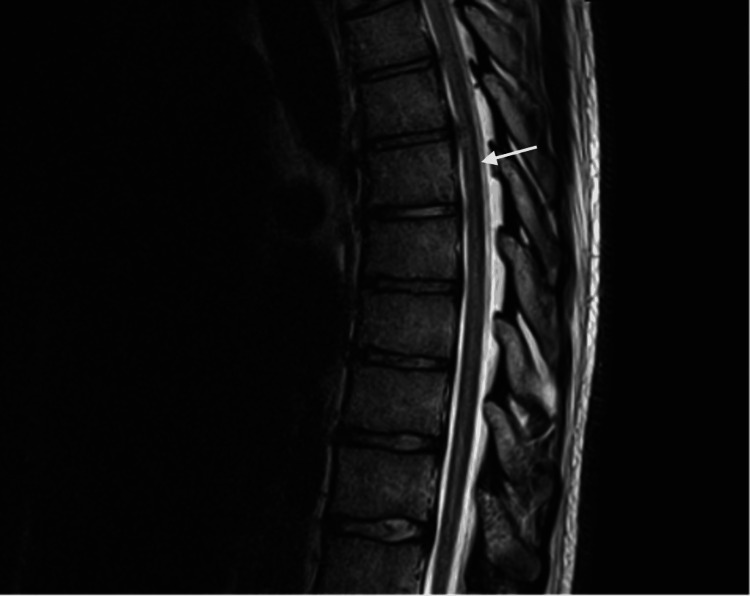
Case 2: Sagittal view of the thoracic spine The image shows posterior midline superficial siderosis from T4-T5 to T10-T11 (arrowed).

**Figure 6:**
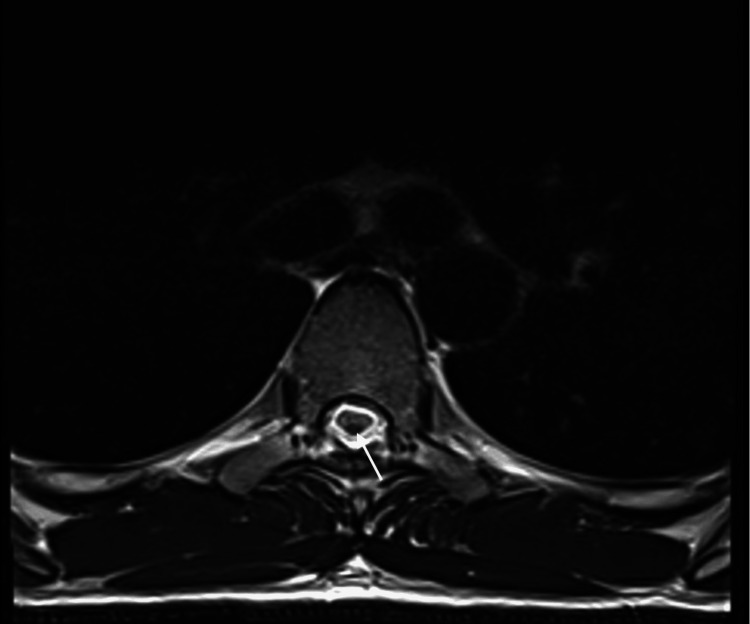
Case 2: Transverse view of the thoracic spine The image shows posterior midline superficial siderosis (arrowed). It remains unclear why the location of hemosiderin deposition was limited to the posterior midline.

Furthermore, there was posterior midline SS from T4-T5 to T10-T11 (Figures 5-6).

Given the history of a gunshot wound to his thoracic spine resulting in significant trauma, the underlying etiology of SS is suspected to be related to a dura tear at the site of the gunshot wound. Since the patient did not exhibit symptoms indicative of brain involvement, an MRI of the brain was not conducted. A referral to Neurosurgery was initiated, and ongoing symptom monitoring, along with future imaging, including the possibility of a brain MRI, has been planned.

## Discussion

This report presents two cases of iSS, with a diagnosis confirmed through MRI. These cases exhibit distinctive clinical presentations, shedding light on the remarkable heterogeneity of this condition. The first case displayed hemosiderin deposition in the cerebellar vermis and bilateral cerebellar hemispheres, whereas the second case revealed hemosiderin deposits within the thoracic spinal cord. Notably, the etiologies of these two cases differed. The first case had a history of multiple spinal surgeries, further complicated by epidural fluid collection at the thoracic level. In contrast, the second case experienced traumatic thoracic SCI resulting from a gunshot injury, further complicated by the development of a syrinx. Our detailed documentation of these patients’ clinical trajectories, MRI findings, and the employed management strategies contributes valuable insights to the existing literature, enriching our understanding of iSS.

To date, only a few studies have explored the prevalence of iSS. Pichler et al. conducted a population-based study involving 1412 MRI brain images, identifying two participants with iSS [1]. In a study by Friedauer et al., 51 iSS were found among 97,733 screened MRI images [3]. Most case reports involving spinal dural defects typically present with intracranial hemosiderin deposition [8-10]. Notably, the cortical and cerebellar surfaces are preferentially involved, and the involvement of the spinal cord is rather scarce [1]. There is a lack of prevalence data specifically related to spinal iSS, and the limited information available is primarily derived from case reports [11]. To our knowledge, the case we reported, involving iSS at the spinal cord due to a gunshot wound, marks the first case report of ballistic injury-related spinal iSS.

Patients with iSS commonly exhibit a spectrum of progressive neurological symptoms. These may include hearing loss, imbalance, truncal ataxia, pyramidal signs, cognitive dysfunction, bladder and bowel disturbances, symptoms of intracranial hypotension, symptoms associated with archnoiditis (such as lower back pain and sciatica pain), dysphagia, hydrocephalus, olfactory dysfunction, ageusia, myoclonus, seizure, visual disturbances, cranial nerve palsies, and paraparesis/quadriparesis [6]. It is noteworthy that it can also be incidentally identified in asymptomatic patients [12]. With the advancement and wide availability of MRI, the diagnosis of SS has become readily feasible, even before the manifestation of clinical symptoms.

In our two cases, the clinical presentations were notably divergent. The first case presented with the classical cluster of symptoms associated with iSS, including ataxia, hearing loss, and cognitive dysfunction. In contrast, the second case presented with lower back pain and lower limb spasticity. The distinct clinical manifestations in these cases are likely attributed to the different anatomical locations of hemosiderin deposition. Given the variability in the clinical presentation of iSS, iSS can potentially be underrecognized or underdiagnosed. Therefore, it is critical that patients with risk factors, who present with clinical symptoms, should undergo MRI for further evaluation to confirm the diagnosis.

The most commonly identified etiology for SS is a spinal (and less commonly, intracranial) dural defect, typically manifesting as a dural tear [4,13]. Persisting cerebrospinal fluid (CSF) leaks are frequently observed in patients with iSS [3,13]. Existing literature indicates that the underlying dural abnormality can be a result of previous cranio-spinal trauma, such as brachial plexus avulsions, previous spinal surgeries, or disc herniations [6,8,9]. Tumors and vascular malformations have also been reported in association with SS [4,14], while in some cases, the cause remained unknown [6]. In the two cases presented here, the likely cause of iSS is related to a spinal dural defect. The first case underwent numerous surgeries for spinal cord untethering and duraplasty. Her MRI revealed thoracic epidural fluid collection. In some cases, fluid collections may appear extradural but are often found to be the source of bleeding [3]. The second case experienced a traumatic thoracic SCI due to a gunshot wound, managed conservatively. Hemosiderin deposition was identified along his thoracic spine, corresponding to the site of his previous gunshot wound. The gunshot injury likely resulted in a dural tear, leading to slow extravasation and subsequent hemosiderin deposition on the surface of the thoracic spinal cord. When SS is observed in cases with the context of a spinal epidural fluid collection or previous spinal trauma, consideration of a dural defect is crucial. MRI of the entire neuraxis is the preferred diagnostic method and initial workup to identify the dural defect [4]. In Case 2, an MRI of the brain was omitted as the patient had no symptoms to suggest cortical, brain stem, or cerebellar involvement, with the dural tear presumably stemming from a previous spinal gunshot wound.

Historically, the diagnosis of SS was often confirmed through autopsy or intra-operative procedures [6]. However, with the widespread availability of MRI, SS is now typically diagnosed by characteristic MRI findings [4,6]. These findings include T2-weighted sequences revealing a marginal rim of hypointensity around the ventral and dorsal surfaces of the brainstem and, at times, the spinal cord. Additionally, gradient-recalled echo and SWI are more sensitive and specific than T2-weighted MRI for identifying haemosiderin deposits [6]. In our cases, the MRI finding in the first case revealed extensive linear and punctate foci of susceptibility involving the cerebellar vermis and bilateral cerebellar folia, while the second exhibited SS along the posterior midline from T4-T5 to T10-T11. These distinctive MRI features strongly support the diagnosis of iSS.

The management of SS entails underlying and addressing the underlying cause, along with symptomatic management. Treatment of dural defects is important to minimize the impact of irreversible neurological impairment [15]. In both cases, consultations with Neurosurgery were sought to explore the potential for surgical intervention. The first case, having undergone numerous spinal surgeries, opted for conservative management. Due to relatively minimal symptoms, the second case is still on close monitoring without surgery. Previous literature often recommends surgery once a dural tear is identified, even in relatively asymptomatic patients [4]. Chelation therapy is not routinely administered [6]. Observational studies on SS receiving chelation have shown decreased hemosiderin deposition on MRI, but clinical outcomes varied [16]. It is vital to emphasize the importance of a multidisciplinary team in managing these cases. In our presented cases, Rehabilitation Medicine, Physical Therapy, and Occupational Therapy played essential roles in monitoring neurological and functional progression, providing targeted therapy to address specific impairments. In the first case, Audiology was consulted to address hearing impairment and the application of a hearing aid. Additionally, a Neuropsychology referral was made for formal cognitive assessment and follow-up. The prognosis of SS is variable, contingent on the extent of irreversible damage caused by hemosiderin deposition.

It is essential to acknowledge the limitations of this case series. Firstly, the underlying etiology of SS is not definitively confirmed. While the clinical findings and trajectories strongly indicate a spinal dural defect, the gold standard for confirming the diagnosis is through surgery. As both cases were managed conservatively, the diagnoses remained uncertain. Secondly, there is a constraint in the follow-up period, limiting the ability to monitor the progression of the disease. These limitations should be taken into account when interpreting the presented cases and when applying insights for future cases.

## Conclusions

In conclusion, iSS is a rare condition that typically presents with gait ataxia, hearing impairment, and cognitive dysfunction. Additionally, myelopathy-related symptoms, back pain, and worsening of spasticity may manifest. iSS is strongly associated with spinal dural defects. In cases where individuals with known spinal dural defects experience progressive worsening of neurological symptoms, MRI imaging with iron-sensitive sequences is essential to confirm the diagnosis. The management highlights the significance of a multidisciplinary team approach.
